# The Effect of Renal Dysfunction on Circulating Sclerostin Level in Patients with Type 2 Diabetes

**DOI:** 10.1155/2014/715908

**Published:** 2014-06-26

**Authors:** Se Hwa Kim, Soo Young Yoon, Sung-Kil Lim, Yumie Rhee

**Affiliations:** ^1^Division of Endocrinology, Department of Internal Medicine, Kwandong University College of Medicine, 100-25 Simgok-ro, Seo-gu, Incheon 404-834, Republic of Korea; ^2^Division of Nephrology, Department of Internal Medicine, Kwandong University College of Medicine, 100-25 Simgok-ro, Seo-gu, Incheon 404-834, Republic of Korea; ^3^Department of Internal Medicine, Severance Hospital, Endocrine Research Institute, Brain Korea 21 Project for Medical Science, Yonsei University College of Medicine, 50-1 Yonsei-ro, Seodaemun-gu, Seoul 120-752, Republic of Korea

## Abstract

*Objective*. Sclerostin is a Wnt inhibitor produced specifically by osteocytes. However, it is not currently clear whether renal dysfunction has an effect on circulating sclerostin level in patients with type 2 diabetes. The aim of the study was to evaluate this relationship. *Design and Patients*. We conducted a cross-sectional observational study of 302 type 2 diabetic patients with or without chronic kidney disease. Serum sclerostin level was analyzed by ELISA, and renal function was assessed by estimated glomerular filtration rate (eGFR) using chronic kidney disease epidemiology collaboration (CKD-EPI) equation. *Results*. There was a strong correlation between sclerostin level with renal function presented as serum creatinine (*r* = 0.745, *P* < 0.001) and eGFR (*r* = −0.590, *P* < 0.001). Serum sclerostin level was significantly higher in patients with CKD-G3 stage than those with CKD-G1/2 stages after adjusting for age, sex, and BMI (*P* = 0.011). Patients with CKD-G4/5 stages had dramatically increased level of circulating sclerostin. Multiple regression analyses found that age, sex, and eGFR were independent determining factors for circulating sclerostin level. *Conclusion*. Our data showed that serum sclerostin levels start to increase in diabetic patients with CKD-G3 stage. Further studies are needed to establish the potential role of elevated sclerostin in diabetic patients with CKD.

## 1. Introduction

Sclerostin is a glycoprotein secreted almost exclusively by osteocytes. It negatively regulates bone formation by binding to low-density lipoprotein receptor-related proteins (LRPs) 5/6 and by antagonizing the Wnt/*β*-catenin signaling pathway [1–3]. Circulating sclerostin concentration has been found to be higher in men than in women [4]. Sclerostin concentration positively correlates with age, body mass index (BMI), and bone mineral density (BMD) [5]. Recent report showed that diabetic patients had higher sclerostin level than nondiabetic subjects [6].

There are few data regarding the effects of liver or kidney function on serum sclerostin level. Previously, we reported that sclerostin level was higher in patients with liver cirrhosis than in healthy controls and correlated with markers of liver dysfunction such as albumin [7]. With regard to renal function, Cejka et al. [8] found that patients with chronic kidney disease (CKD) stage 5 on dialysis had higher sclerostin levels than those without CKD and suggested elevated sclerostin may play a role in renal osteodystrophy. Also, recent report found that serum sclerostin levels increase as estimated glomerular filtration rate (eGFR) decreases in patients with CKD [9].

However, so far it was unknown whether this finding applies to the patients with type 2 diabetes. In the present study, we assessed serum sclerostin level according to renal function in patients with type 2 diabetes.

## 2. Patients and Methods

### 2.1. Study Population

Our study was a cross-sectional study that included type 2 diabetic patients with or without CKD. A total of 302 diabetic men and women who visited the endocrinology or nephrology clinic were recruited from Myongji Hospital or Severance Hospital from March 2010 to September 2011. The study was approved by the Institutional Review Board of Kwandong University College of Medicine or Yonsei University College of Medicine. All the patients in the study provided written informed consent.

### 2.2. Laboratory Measurements

Samples of venous blood were taken in the morning after fasting overnight. Sera were stored at −80°C until examination. Fasting plasma glucose, glycated hemoglobin (HbA1C), AST, ALT, and creatinine (Cr) were measured using standard automated laboratory techniques. Estimated glomerular filtration rate (eGFR) was calculated using chronic kidney disease epidemiology collaboration (CKD-EPI) creatinine equation. Patients were divided into three groups by CKD stages according to decreased degrees of GFR (CKD-G1/2, G3, and G4/5 stages). Subjects with eGFR greater than 90 mL/min/1.73 m^2^ were categorized as CKD-G1. G2 denotes eGFR 60~89 mL/min, G3 30~59 mL/min, G4 15~29 mL/min, and G5 < 15 mL/min/1.73 m^2^. Serum sclerostin was measured using a quantitative sandwich ELISA method (Biomedica Co., Vienna, Austria). The intra-assay and interassay coefficients of variations (CVs) were 4–6% and 5–7%, respectively. Serum intact parathyroid hormone (PTH) was measured by immunoradiometric assay (Biosource, Nivelles, Belgium, with an intra-assay CV 2.7% and interassay CV 3.5%).

### 2.3. Statistical Analysis

**Figure 1:**
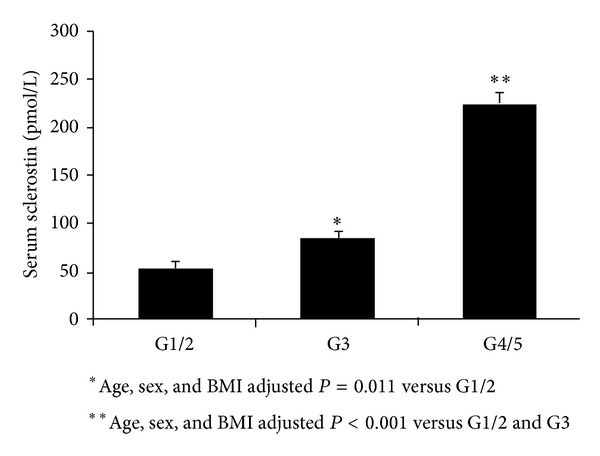
Serum sclerostin levels according to CKD groups in patients with type 2 diabetes. Values are expressed as mean ± SE.

Statistical analysis was performed with SPSS version 11.0. The values were presented as mean ± SD or median and interquartile range (Table 1) or mean ± SE (Figure 1). Pearson correlation analysis was used to assess the correlations between serum sclerostin and other parameters. We performed multivariate linear regression analysis to investigate the association between eGFR and serum sclerostin. Age, sex, BMI, HbA1C, serum calcium, phosphorus, PTH, and duration of diabetes were included in regression analysis. Univariate analysis of covariance (ANCOVA) was used to evaluate the differences of sclerostin level in men and women between the different CKD groups after adjusting for age, sex, and BMI. A* P* value < 0.05 was considered statistically significant.

## 3. Results

### 3.1. Characteristics of the Study Patients

The clinical and biochemical characteristics of the study patients are shown in Table 1. The average age was 67.4 ± 7.3 years, with a range of 50–88. The mean eGFR was 53.9 ± 26.8 mL/min/1.73 m^2^. Median sclerostin level was 64.4 pmol/L (interquartile range 45.4–91.1).

Forty-nine percent of patients had eGFR ≥60, 30% had eGFR 30–59, and 21% had eGFR <30 mL/min/1.73 m^2^ (Table 1). Men had significantly higher sclerostin levels than women after adjusting for age, BMI, and eGFR (121.4 ± 6.8 versus 76.1 ± 6.5 pmol/L, *P* < 0.001).

### 3.2. Correlation of Serum Sclerostin with Renal Function

Patients were classified into three groups according to their eGFR categories (G1/2 versus G3 versus G4/5). We compared serum sclerostin levels according to the groups using ANCOVA. Serum sclerostin level was significantly higher in patients with G4/5 or G3 than those with G1/2 after adjusting for age, sex, and BMI (225.5 ± 10.9 versus 84.9 ± 9.2 versus 54.6 ± 6.9 pmol/L) (Figure 1).

There was a strong correlation between sclerostin level and renal function, presented as serum Cr and eGFR in the entire cohort (Cr; *r* = 0.745, eGFR; *r* = −0.590, resp., *P* < 0.001) (Table 2). Multiple regression analysis showed that eGFR was an independent determining factor for circulating sclerostin level in the entire cohort (standardized beta coefficients = −0.659, 95% CI −4.8, −2.4) (Table 3).

### 3.3. Correlation between Serum Sclerostin, PTH, Calcium, and Phosphate

Serum PTH was measured only in a small proportion of the subjects (*n* = 114). Univariate analysis showed positive correlation of PTH with serum sclerostin in the entire cohort or patients with CKD-G3~5. However, serum sclerostin was not correlated with PTH in patients with CKD-G1/2 (Table 2). Multiple regression analysis found that PTH was not an independent factor for serum sclerostin in the entire cohort or CKD subgroups.

Univariate analysis revealed that there were significant correlations between serum sclerostin and phosphate (*r* = 0.452, *P* < 0.001) or calcium (*r* = −0.343, *P* < 0.001) in the entire cohort, but these relationships disappeared in patients with G1/2 (*r* = −0.049, *P* = 0.679 for phosphate, *r* = 0.051, *P* = 0.662 for calcium). There was a weak positive correlation between serum phosphate and sclerostin in the entire cohort (*β* = 0.153, *P* = 0.059), which did not persist when different CKD groups (*β* = 0.128, *P* = 0.474 for CKD-G1/2 group, *β* = 0.066, *P* = 0.517 for CKD-G3~5 group) were analyzed separately in the multiple regression analysis.

### 3.4. Correlation of Serum Sclerostin with BMI and Duration of Diabetes

BMI showed negative correlation with serum sclerostin in all patients (*r* = −0.157, *P* = 0.007), but it positively correlated with sclerostin in patients with G1/2 (*r* = 0.213, *P* = 0.009) (Table 2). The negative correlation of BMI with sclerostin only remained in patients with CKD-G3~5 after multivariate analysis (*r* = −0.196, *p* = 0.033) (Table 3).

Duration of diabetes significantly and positively was correlated with serum sclerostin in the entire cohort (*r* = 0.381, *P* < 0.001) (Table 2). However, duration of diabetes was not an independent factor for circulating sclerostin in multiple regression analysis (*β* = −0.069, *P* = 0.372). Multiple regression analysis has shown that eGFR, age, and sex were independently associated with serum sclerostin in the entire cohort (Table 3).

## 4. Discussion

This cross-sectional study found that circulating sclerostin level was significantly higher in type 2 diabetic patients with CKD-G3 or G4/5 stage than those with G1/2 stage. Also, we found that eGFR, age, and sex were independently associated with circulating sclerostin.

Previously, Cejka et al. found that patients with CKD stage 5 on dialysis showed higher sclerostin levels than patients without CKD (2055 ± 1239 pg/mL in CKD 5 patients on dialysis; 480 ± 150 pg/mL in premenopausal women; 1160 ± 380 pg/mL in postmenopausal women) [8, 10]. They reported that sclerostin showed a significant negative association with parameters of bone turnover such as activation frequency and bone formation rate (BFR)/bone surface (BS) in stage 5D CKD patients. They also found a significant association between sclerostin and osteoblast number [8].

Our study found serum sclerostin levels were 1.5 times higher in diabetic patients with CKD-G3 stage than in those with G1/2 after adjusting for age, sex, and BMI. Moreover, serum sclerostin levels were 4 times higher in patients with CKD-G4/5 stage compared with those with G1/2. Our results were consistent with the recent report by Sabbagh et al. [11]. They reported that repression of osteocytes Wnt/*β*-catenin signaling and increased expression of sclerostin occurred in early stage of CKD in a genetic model of mice. They suggested that repression of the Wnt/*β*-catenin pathway is an early event in the progression of renal osteodystrophy. Interestingly, recent report found that increased sclerostin levels in CKD patients were not due to decreased renal elimination. On the contrary, increased circulating sclerostin is the result of increased sclerostin production in uremic patients [12]. The mechanisms underlying increased production of sclerostin in CKD are yet unknown. Our results and recent other reports suggest that higher circulating sclerostin due to increased production might have a role of decreased bone quality in diabetic patients with early CKD.

The effects of PTH on bone are mediated, at least partly, through inhibition of sclerostin expression [10, 13–15]. Many studies, but not all, have shown inverse correlation between serum sclerostin and PTH level [8, 10, 15–17]. Mirza et al. [10] reported that serum sclerostin levels negatively correlated with PTH in postmenopausal women without CKD. Cejka et al. [8] also showed significant inverse correlation between sclerostin and PTH in 60 patients with stage 5 CKD on dialysis. Our study found that there was a positive correlation between sclerostin and PTH in the univariate analysis of the entire cohort or patients with CKD-G3~5, although this correlation did not persist in the multiple regression analysis. The cause of inconsistent results between our study and other reports is unknown. One possibility is that renal failure leads to skeletal resistance to PTH, and decreases in PTH signalling activity might result in increased production of sclerostin in CKD patients. Another explanation is that as the major determinant of serum PTH and sclerostin is GFR this might have largely overridden the results in the present study.

With regard to correlation of serum phosphate with sclerostin, few reports and results are conflicting so far. Pelletier and colleagues [9] reported that serum phosphate was independently associated with sclerostin level in 90 adult patients with CKD. Recent report found that dietary phosphate stimulated bone sclerostin expression independently of PTH in a model of CKD-adynamic bone disease (ABD) [18]. On the other hand, Cejka et al. [12] reported that there was no association between serum sclerostin and fractional excretion of phosphate in a multivariate analysis. They suggested that rises in phosphate and sclerostin levels in CKD patients occur simultaneously but are not necessarily mechanistically linked. In the present study, there was a weak correlation between serum phosphate and sclerostin in the entire cohort (*P* = 0.059), which did not persist when different CKD groups were analyzed separately in the multiple regression analysis.

We found that serum sclerostin was significantly associated with duration of diabetes or HbA1C in the univariate analysis. However, they were not independent factors for circulating sclerostin in the multiple regression analysis. Our results are inconsistent with previous report by Garcia-Martin et al. [6]. They reported that serum sclerostin was correlated with duration of diabetes and glycated hemoglobin in patients with type 2 diabetes. However, they adjusted for only age to investigate the association between duration of diabetes and sclerostin. In the present study, serum sclerostin was significantly associated with duration of diabetes after adjusting for age, sex, and eGFR (*β* = 0.192, *P* < 0.001, data not shown), which disappeared after adjusting further confounders.

We also observed that men had significantly higher sclerostin levels than women after adjusting for age, BMI, and renal function. This finding is consistent with previous data from other groups. Mödder et al. [4] reported that men had higher circulating sclerostin levels than women in a population-based sample. Kirmani et al. [19] demonstrated that serum sclerostin levels were higher in boys aged 6–21 years, compared with girls of the same age range. They suggested that the gender difference in sclerostin level appears to be established during puberty. Another group found that age and serum estradiol (E2) levels were determinants of serum sclerostin level in healthy pre- and postmenopausal women [20]. Therefore, it is plausible that lower estrogen level as well as a larger skeleton may explain the higher circulating sclerostin level in men compared with women. However, recent observational study did not find any difference on sclerostin level between men and women after adjusting for age, bone mineral content, physical activity, BMI, and renal function [5]. The reason for these different results is not clear.

With regard to age, Ardawi et al. [20] reported that serum sclerostin level increases significantly with age in healthy pre- and postmenopausal women. Serum sclerostin level increases over life by an average of 3.7-fold (*P* < 0.0001). They suggested that increased sclerostin production by osteocytes may be involved in the age-related impairment of bone formation. Intriguingly, age was negatively correlated with serum sclerostin in the multiple regression analysis of our study. We cannot explain the reason, but it is possible that our study subjects had wide range of renal function including CKD5 and renal function acts as a strong determining factor on circulating sclerostin level, which leads to having an effect on the relationship between sclerostin and age.

This study has several limitations. First, this is a cross-sectional observational study that demonstrated associations, not causal relationships. Second, serum PTH levels were measured in a small proportion of study patients and may affect the statistical power of our study. Therefore, we tried to overcome this by adding PTH to the multivariate analysis.

In conclusion, we have shown that circulating sclerostin level negatively correlated with eGFR in diabetes patients with wide range of renal function. Also, serum sclerostin levels start to increase in diabetic patients with CKD-G3 stage. Further, studies are needed to establish the potential role of elevated sclerostin in diabetic patients with early CKD.

## Figures and Tables

**Table 1:** Clinical and biochemical characteristics of patients (*n* = 302).

Characteristics	Values
Age (years)	67.4 ± 7.3
Men/women (*n*/*n*)	143/159
Body mass index (kg/m^2^)	24.8 ± 3.1
Duration of diabetes (years)	14.6 ± 9.1
Fasting glucose (mg/dL)	151.4 ± 59.4
HbA1C (%)	7.6 ± 1.4
Aspartate transaminase (mg/dL)	23.2 ± 11.5
Alanine transaminase (mg/dL)	21.4 ± 13.7
Serum calcium (mg/dL) [*n* = 174]	9.2 ± 0.6
Serum phosphorus (mg/dL) [*n* = 174]	4.0 ± 0.9
Parathyroid hormone (pg/mL) [*n* = 114]	52.9 [27.5–170.8]
25(OH)D (ng/dL)	35.5 ± 22.7
Serum creatinine (mg/dL)	2.3 ± 2.9
Serum sclerostin (pmol/L)	64.4 [45.4–91.1]
eGFR (ml/min/1.73 m^2^)	53.9 ± 26.8
CKD-eGFR categories	
G1/2 (≥60 ml/min/1.73 m^2^)	149 (49.3%)
G3 (30~59 ml/min/1.73 m^2^)	90 (29.8%)
G4/5 (<30 ml/min/1.73 m^2^)	63 (20.9%)

Data are shown as mean ± SD or median [interquartile range] as appropriate.

**Table 2:** Univariate correlations between serum sclerostin level and various parameters.

Variables	Group with CKD-G1/2		Group with CKD-G3~5		Total group	
	*r *	*P* value	*r *	*P* value	*r *	*P* value
Age	0.031	0.711	−0.219	0.007	−0.048	0.403
Male sex	0.481	<0.001	0.211	0.009	0.158	0.006
Body mass index	0.213	0.009	−0.240	0.004	−0.157	0.007
HbA1C	−0.244	0.005	−0.162	0.108	−0.129	0.051
Duration of diabetes	0.019	0.834	0.389	<0.001	0.381	<0.001
Serum creatinine	0.526	<0.001	0.708	<0.001	0.745	<0.001
eGFR	−0.219	0.007	−0.574	<0.001	−0.590	<0.001
Calcium	0.051	0.662	−0.302	0.002	−0.343	<0.001
Phosphorus	−0.049	0.679	0.391	<0.001	0.452	<0.001
PTH	−0.265	0.136	0.383	<0.001	0.485	<0.001

**Table 3:** Multiple regression analysis to identify factors associated with serum sclerostin level.

Variables	Group with CKD-G1/2		Group with CKD-G3~5		Total group	
	*β*	*P* value	*β*	*P* value	*β*	*P* value
	*R* ^2^ = 0.586		*R* ^2^ = 0.868		*R* ^2^ = 0.809	
Age	0.010	0.958	−0.205	0.035	−0.214	0.004
Male sex	0.506	0.009	0.240	0.004	0.172	0.008
Body mass index	0.224	0.296	−0.196	0.033	−0.135	0.074
HbA1C	−0.257	0.164	−0.065	0.385	−0.026	−0.415
Duration of diabetes	0.003	0.987	−0.042	0.621	−0.069	0.372
Calcium	0.146	0.796	−0.002	0.983	−0.118	0.128
Phosphorus	0.128	0.474	0.066	0.517	0.153	0.059
PTH	−0.279	0.112	−0.193	0.141	0.058	0.577
eGFR	−0.166	0.435	−0.868	<0.001	−0.659	<0.001
